# Nested PCR followed by NGS: Validation and application for HPV genotyping of Tunisian cervical samples

**DOI:** 10.1371/journal.pone.0255914

**Published:** 2021-08-11

**Authors:** Monia Ardhaoui, Emna Ennaifer, Anna Christina De Matos Salim, Flávio Marcom Gomez, Thalja Laasili, Med Samir Boubaker, Ikram Guizani

**Affiliations:** 1 Department of Molecular Epidemiology and Experimental Pathology, Institut Pasteur de Tunis, University of Tunis El Manar, Tunis, Tunisia; 2 Department of Human and Experimental Pathology, Institut Pasteur de Tunis, Tunis, Tunisia; 3 Genomics and Computational Biology Group, Fiocruz—Research Center Renê Rachou, Belo Horizonte, Minas Gerai, Brazil; Universidad de Chile, CHILE

## Abstract

The most used methodologies for HPV genotyping in Tunisian studies are based on hybridization that are limited to a restricted number of HPV types and to a lack of specificity and sensitivity for same types. Recently, Next-Generation sequencing (NGS) technology has been efficiently used for HPV genotyping. In this work we designed and validated a sensitive genotyping method based on nested PCR followed by NGS. Eighty-six samples were tested for the validation of an HPV genotyping assay based on Nested-PCR followed by NGS. These include, 43 references plasmids and 43 positive HPV clinical cervical specimens previously evaluated with the conventional genotyping method: Reverse Line Hybridization (RLH). Results of genotyping using NGS were compared to those of RLH. The analytical sensitivity of the NGS assay was 1GE/μl per sample. The NGS allowed the detection of all HPV types presented in references plasmids. On the clinical samples, a total of 19 HPV types were detected versus 14 types using RLH. Besides the identification of more HPV types in multiple infection (6 types for NGS versus 4 for RLH), NGS allowed the identification of HPV types that were not detected by RLH. In addition, the NGS assay detected newly HPV types that were not described in Tunisia so far: HPV81, HPV43, HPV74, and HPV62. The high sensitivity and specificity of NGS for HPV genotyping in addition to the identification of new HPV types may justify the use of such technique to provide with high accuracy the profile of circulating types in epidemiological studies.

## Introduction

High risk Human Papillomavirus (HR-HPV) infection is known to be the leading cause of Cervical cancer (CC) [1, 2]. CC remains the second most common cause of death from gynecological cancer in women worldwide (Globocan 2020) (https://www.uicc.org/news/globocan-2020-new-global-cancer data). In Tunisia, it is the fourth cause of mortality in women with 185 deaths each year (https://www.uicc.org/new-global-cancer-data-globocan-2020).

Two hundred HPV genotypes have been characterized to date and more than 40 types have been reported to infect the female genital tract [2, 3]. There are many commercial methods available for HPV genotyping which are mainly based on PCR and hybridization [4, 5]. However, this approach can recognize sufficiently similar HPV types, allowing the detection of a restricted number of types, and hampering discrimination among nucleotide variants [4, 6, 7]. Next-Generation Sequencing (NGS) has emerged as a powerful tool for HPV genotyping, with the advantage of processing multiple samples at a time [8–12]. Reported NGS studies of HPV infections have shown a good specificity and sensitivity to study viral diversity. The use of this technology could be important to support pilot epidemiological studies and to assess HPV program vaccination effectiveness.

In a previous study, and in order to evaluate HPV types distribution in Tunisian women, we developed a nested PCR based on detection of HPV L1 using PGMY and GP5+/GP6+ primers, followed by Reverse Line Hybridization (RLH) targeting 31 genotypes [13]. This technology, as many commercial methods, has the limits of detecting a restricted number of HPV genotypes especially in cases of multiple infections and low viral load. It showed that 4% of infections were no typed [13]. In order to solve this issue, this study aimed to design and validate a novel HPV genotyping method based on a nested PCR using modified GP primers followed by a Miseq NGS approach.

## Material and methods

The study (study reference: (2014/2A/ONMNE)) was approved by the Ethical committee of Institut Pasteur de Tunis, subscribed on the office of human research protections under the reference IORG001040, and conducted with good clinical practice, ensuring confidentiality and anonymity.

### Specimens

A total of 86 samples were tested for the validation of the HPV genotyping assay based on Nested-PCR followed by NGS. These include, 43 references plasmids (WHO HPV proficiency panel) and 43 positive HPV clinical cervical specimens.

#### WHO HPV proficiency panel

To validate the sensitivity and specificity of the Miseq genotyping approach here reported, 43 samples of the 2014 WHO proficiency study panel were used as reference samples. This panel was composed of purified plasmid DNA in which genomic DNA of different HPV types was cloned and diluted in a background of human placental DNA as previously described [14]. As described in the HPV labnet international proficiency study [14], each panel was composed of 43 samples that contained different amounts of plasmids (5 and 50 GE per 5 μL for HPV16 and HPV18 and 50, and 500 GE per 5 μL for the other high-risk HPV types 31, 33, 35, 39, 45, 51, 52, 56, 58, 59, 66, 68a and 68b, and the low-risk HPV types 6 and 11) and three samples, used as extraction control, that contained HPV positive (HPV 16; SiHa Cervical cancer cells 25/2500 GE/5μl) and HPV negative cell lines (HPV-negative C33A cells). Some samples contained different HPV types in various amounts to mimic multiple infections.

#### HPV positive clinical samples

The forty-three HPV positive samples identified in an earlier study that examined the prevalence of genital HPV infection in the Region of Grand Tunis [13] were used to be genotyped using NGS approach. The description of clinical samples is available in the previous publication [13].

DNA was extracted from a 200μl aliquot of the suspended cell samples. Extraction and quality control by betaglobin PCR, are as previously described [13]. All samples were positive for betaglobin PCR and were further analyzed for HPV detection and genotyping.

### HPV genotyping

#### Genotyping by RLH

All the clinical samples were typed during a previous study using RLH as previously described [13]. Briefly, the technique permits the identification of 31 types using specific probes (HPV6, 11, 16, 18, 26, 31, 33, 34, 35, 39, 40, 42, 44, 45, 51, 52, 53, 54, 55, 56, 57, 58, 59, 66, 68, 69, 70, 73, 82, 83, and 84) that were immobilized on a Biodyne C membrane.

#### Genotyping using Miseq

*First PCR amplification*. The PGMY primers [15] were used to amplify an L1 HPV DNA region in a 50μl reaction as described previously [13]. Then the PCR products were used in a genotyping protocol using GP5+/GP6+ primers as follows.

*Libraries preparation*. The PGMY PCR amplicons were used in a second PCR using custom designed primers. These primers contain the GP5+/6+ sequence and additional 10 bases Illumina adapter sequences (P5/P7). The sequences of primers are represented in S1 Table.

The PCR reaction was conducted in 25 μl mixture contained 2.5μl of first PCR product, 12.5μl Kapa Mix (Kapa Taq Hot Start Mix, TXHRMKB, Roche). PCR cycling parameters were composed of a 3 minutes of initial denaturation at 95°C, followed by 14 amplifications of 30 seconds at 94°C, 1 minute at 55°C and 1 minute at 72, and a final extension step for 5 minutes at 72°C.

Different specific indexes were added to the generated amplicons. The size of the PCR products (~200 pb) was evaluated with the 2100 Bio analyzer system (Cat. no. G2940CA; Agilent Technologies, Santa Clara, CA, USA). They were purified using the Agent court® Apure® XP beads (Cat. no. A63881; Beckman Coulter Genomics, Danvers, MA, USA) following the manufacturer’s instructions. After purification, 96 different primer indexes, using the NextEra XT Index Kit, were added to the end of HPV amplicons. Each amplicon had a different index, which allowed the pooling of all the samples together.

**Fig 1:**
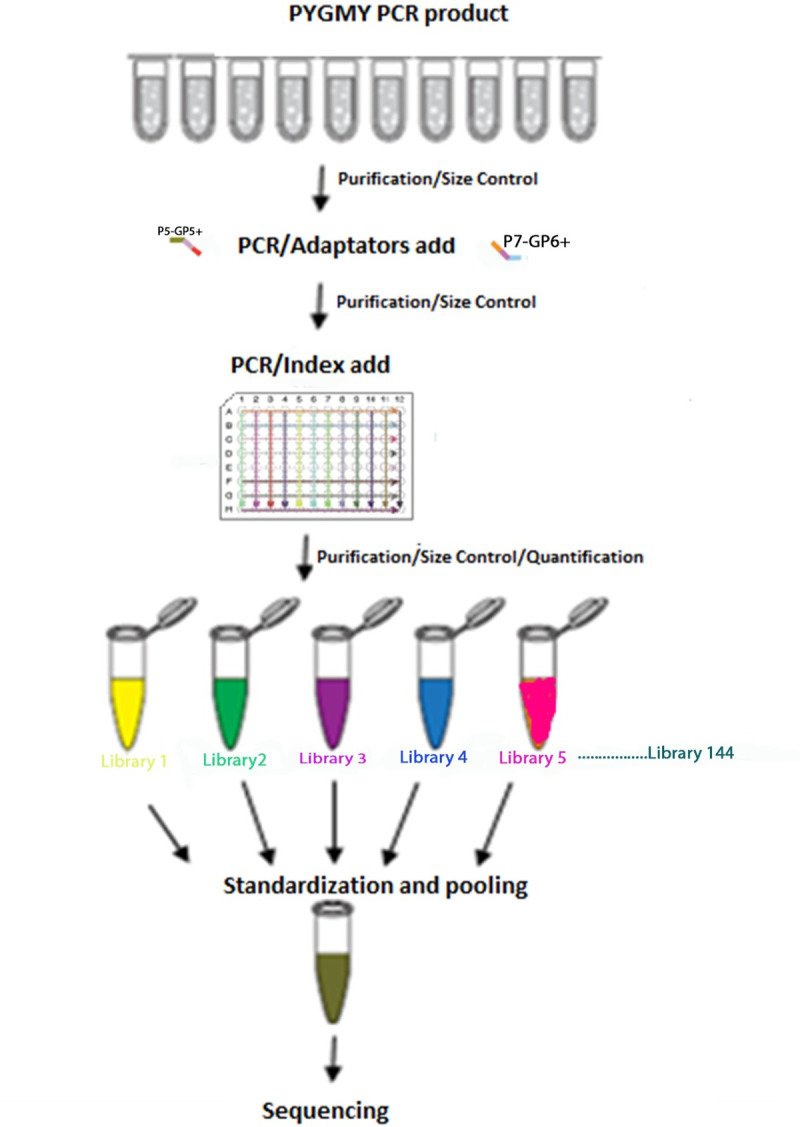
Workflow for library preparation and sequencing. Amplicons generated through a PGMY PCR were amplified using customized primers containing Miseq specific adaptors then indexation and pooling were done before sequencing using Miseq plateform.

Libraries were purified then quantified using Real Time PCR. Pooling was performed at equimolar ratios (4nM) to yield one sequencing sample. In preparation for cluster generation and sequencing, pooled libraries were denatured with NaOH, diluted with hybridization buffer, and then heat denatured before MiSeq sequencing. The sequencing was performed using the Miseq V2 Kit (Fig 1).

*Data analysis*. Specific reads of each sample were identified according to their respective corresponding index. Adaptor sequences and primers were trimmed using Trimmomatic 0.33 tool [16]. Quality control of sequencing data was performed using the FastQC software (http://www.bioinformatics.babraham.ac.uk/projects/fastqc/). The cut-off of read depth was fixed to 30 [17]. Good sequences (Q-Score>30) were firstly mapped into human genome using bowtie, an adapted alignment tool for short reads [18]. Only unmapped reads were assembled to contiguous sequences (contigs) using Trinity software (https://github.com/trinityrnaseq/trinity). We processed only high quality reads having at least 80% of base pairs with base calling accuracy of 99.9%and used stringent assembly parameters in the Trinity assembler.

**Fig 2:**
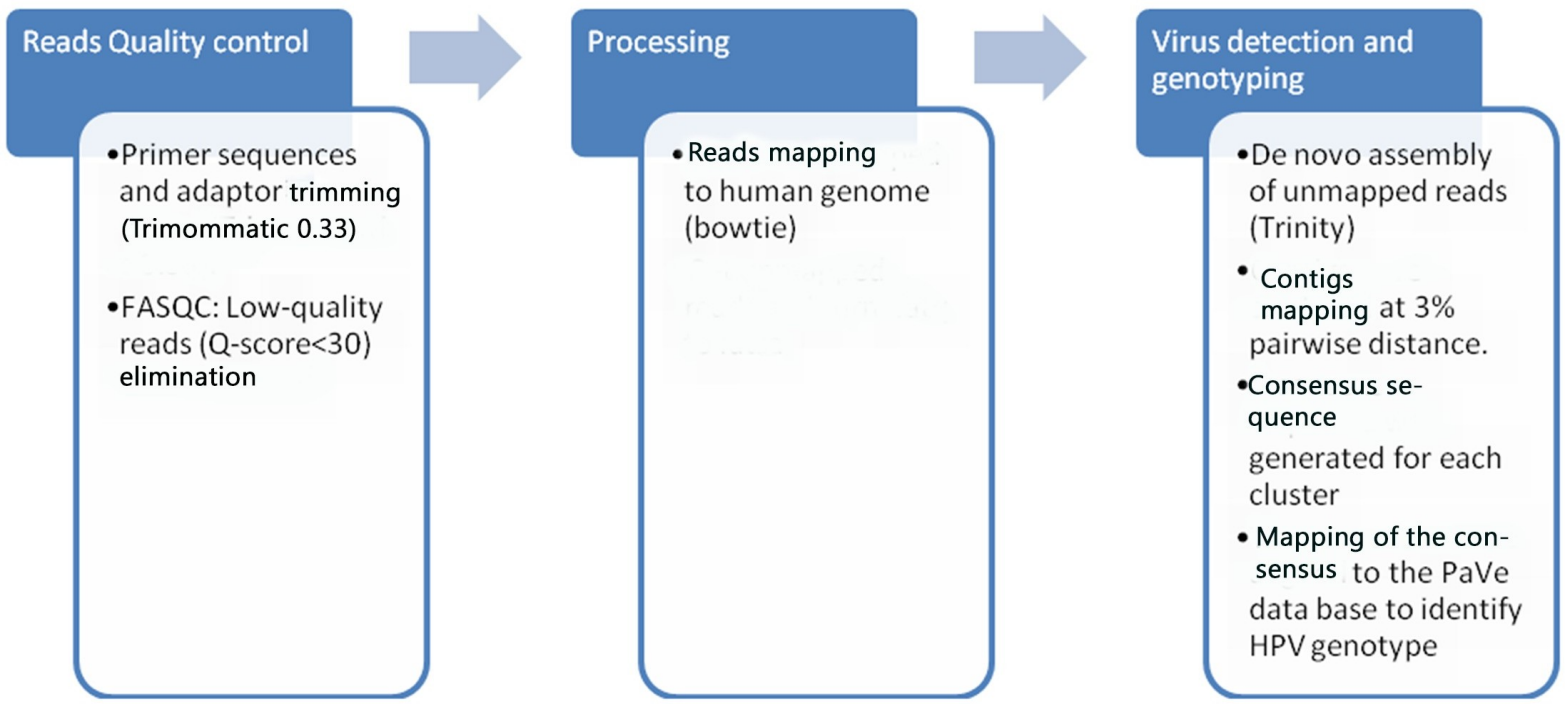
Workflow for data analysis. The designed pipeline for HPV genotyping in this study contains 3 steps: reads quality control to remove bad quality reads, processing of retained reads for mapping into Human genome, and finally in the final set of selected sequences the viruses were detected and genotyped by assembly and contigs mapping to HPV reference genome.

To identify HPV genotypes, contigs were firstly clustered at 3% pairwise distance and a consensus sequence was generated. Consensus sequences were aligned using Bowtie 2 with the L1 region of alpha Papillomavirus reference sequences that were obtained from Papillomavirus Episteme (PaVE) (PaVE; http://pave.niaid.nih.gov) (Fig 2).

### Statistical analysis

HPV genotype frequencies were calculated by simple counts and percentages. Association between HPV infections and read numbers was evaluated using chi-squared test. A p-value <0.05 was considered statistically significant.

The agreement between RLH and NGS was calculated using the Cohen kappa test, interpreted as perfect agreement (κ > 0.81), substantial agreement (κ = 0.61–0.80), moderate agreement (κ = 0.41–0.60), fair agreement (κ = 0.21–0.40), slight agreement (κ = 0.01–0.20) or poor agreement (κ = 0.21–0.40). Statistical counts and tests were performed using R Studio version 1.2.5033 (*http*:*//www*.*rstudio*.*com/**)*.

## Results

### Validation of targeted NGS genotyping by WHO panel

**Fig 3:**
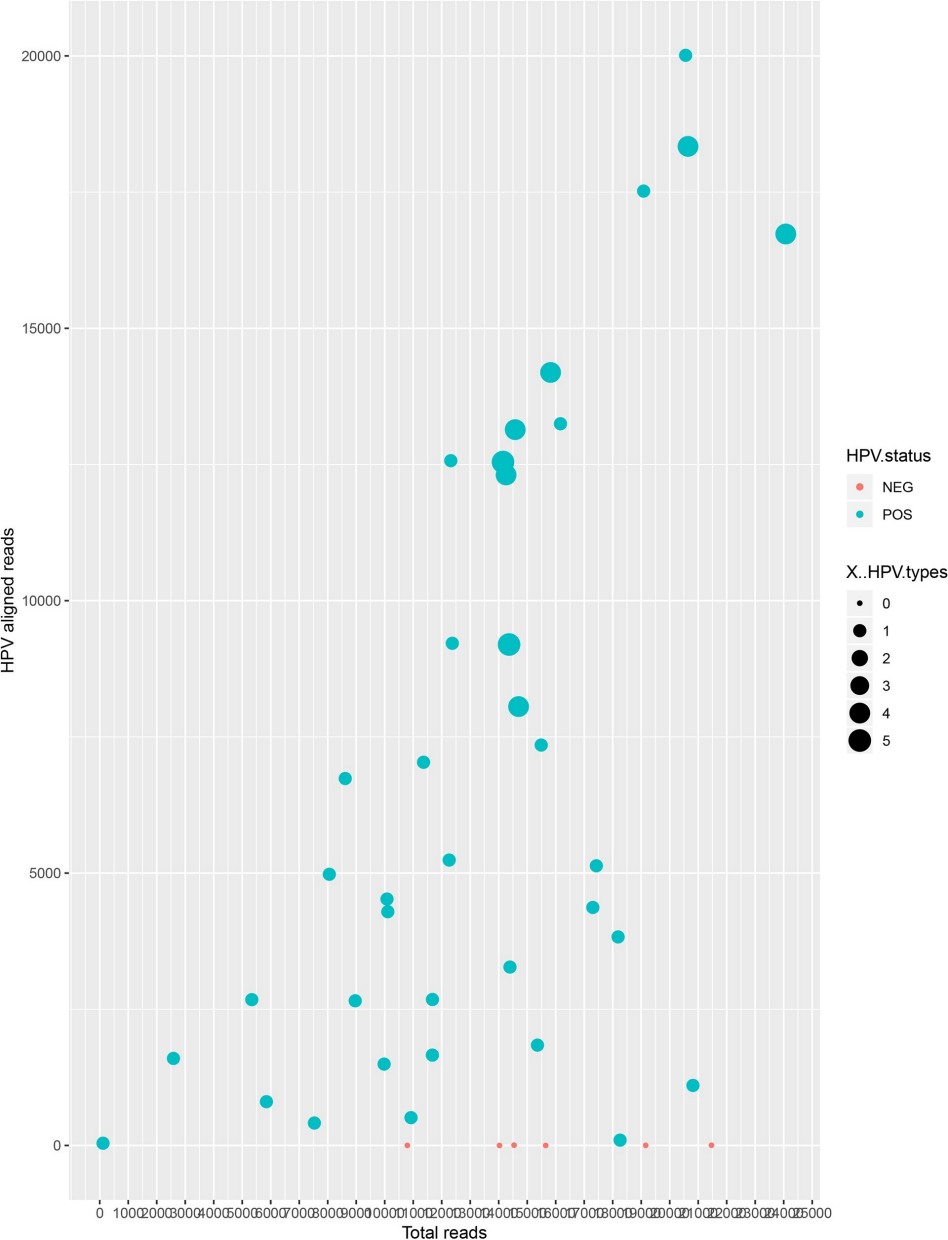
HPV-aligned reads vs. total number of reads according to HPV status and number of HPV types in the samples. There was no difference in total reads number between positive samples (blue dots) and negative samples (red dots). In contrast, alignment of HPV reads depended on the HPV types in each positive sample (p<10^−3^): The aligned HPV reads were more numerous in samples having multiple infections (HPV types number >1): the size of dots indicates the number of HPV types in each types sample.

To validate the genotyping approach using Miseq, the designed protocol was used firstly to analyze the 43 WHO 2014 proficiency plasmids panel. The generated data showed that the total number of reads was approximately similar in both HPV positive and negative plasmids. Median length of HPV reads was 174 nucleotides (nt), ranging between 137 and 180, while the modal length of the reads was 160 nt but without significant differences among samples with different HPV types, nor between samples with HR-HPV and LR-HPV, nor between single and multiple infections. Meanwhile, the HPV aligned reads in HPV positive samples (minimum: 30; maximum: 19166; median: 6335) was different from those of HPV negative (maximum: 6). The greatest number of reads was obtained in multiple infections (HPV types >1) (p<10^−3^) (Fig 3).

To confirm the detected HPV types, a cut-off of 30 reads was used. HPV types in the WHO panel were correctly detected and typed with NGS at different DNA amounts (5 to 2500 GE/5μL). The negative control of this panel was also identified as negative (Panel C). HPV 18 was detected at the level of 5GE/5μl and HPV16 at the level of 25 GE/5μl. All samples showed complete concordance for all types, even with multiple infections containing up to 4 genotypes. No other HPV types from the plasmids were detected (Table 1).

**Table 1:** Proficiency test panel genotyping using NGS technology.

PPTID	GE/5μl	Genotype of the PPT	Identified genotype by NGS
**31**	5	18	18
**3**	50	51	51
**12**	50	35	35
**13**	50	66	66
**14**	50	6/56/58/68	6/56/58/68
**15**	50	11	11
**16**	50	45	45
**17**	50	58	58
**18**	50	68	68
**19**	50	6	6
**22**	50	11/18/31/52	11/18/31/52
**23**	50	31	31
**26**	50	39	39
**32**	50	52	52
**33**	50	59	59
**34**	50	35/39/59/66/68	35/39/59/66/68
**35**	50	18	18
**36**	50	56	56
**38**	50	6	6
**41**	50	16/33/45/51	16/33/45/51
**1**	500	16/33/45/51	16/33/45/51
**4**	500	16	16
**5**	500	39	39
**6**	500	58	58
**7**	500	35/39/59/66/68	35/39/59/66/68
**8**	500	68	68
**9**	500	51	51
**10**	500	31	31
**21**	500	66	66
**24**	500	56	56
**27**	500	6/56/58/68	6/56/58/68
**28**	500	11	11
**29**	500	52	52
**30**	500	11/18/31/52	11/18/31/52
**39**	500	59	59
**42**	500	33	33
**43**	500	35	35

PPT, proficiency panel test.

### Genotyping of positive HPV clinical samples by Miseq

Forty-three HPV positive clinical samples were genotyped using the designed NGS protocol. These samples were firstly genotyped by RLH protocol in a previous study. The results showed that 12 of the 43 samples (4%) had unknown HPV, and a total of 14 types were identified [13].

**Fig 4:**
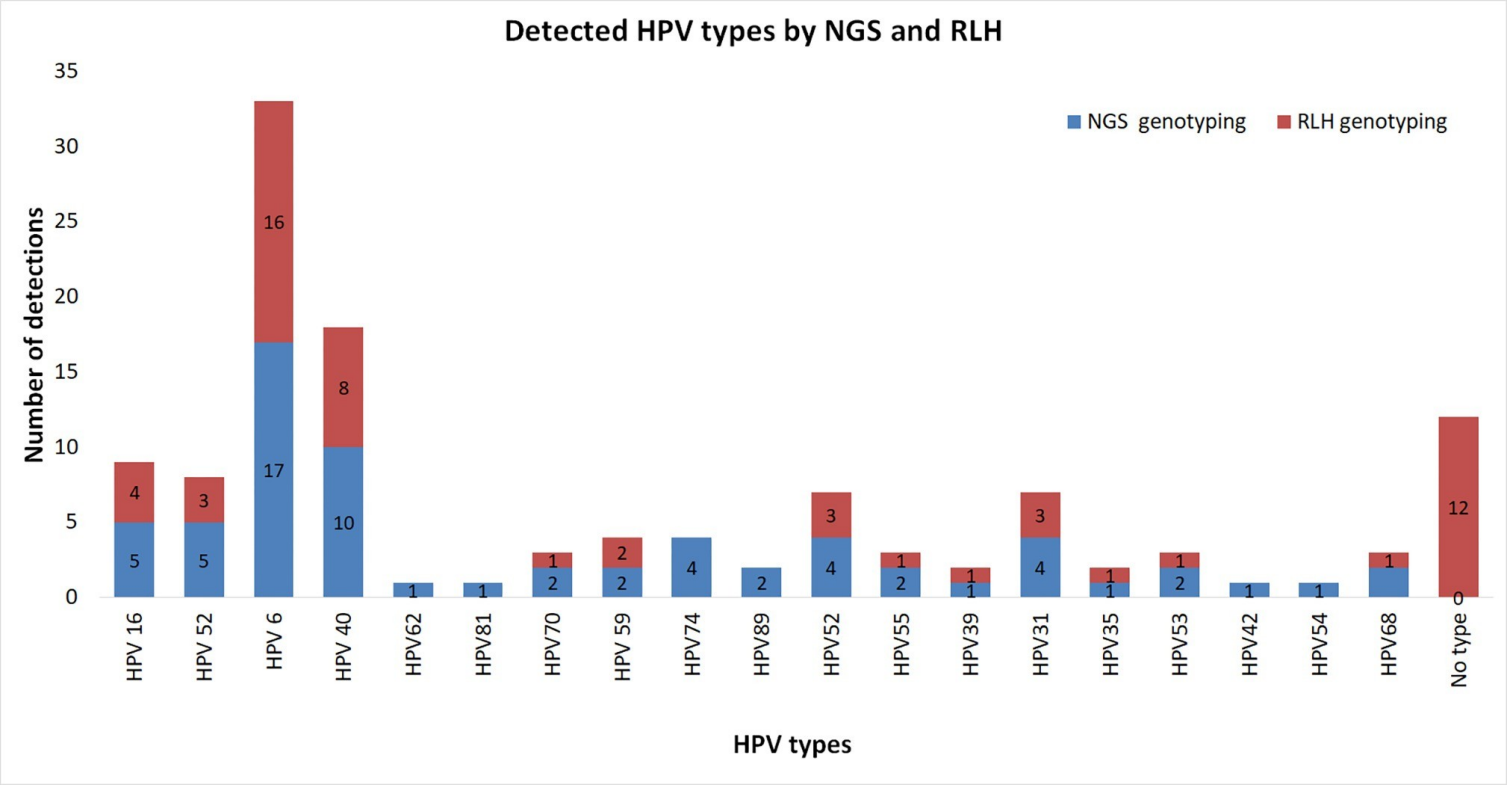
Detected HPV types by NGS and comparison with RLH.

Using the designed NGS genotyping assay, a cut-off of 30 reads was used to confirm the identified HPV type in each sample. As results, all samples were typed and a total of 19 types were identified in addition to the detection of new HPV types; HPV81, HPV43, HPV74, and HPV62 (Fig 4). Single infections and multiple infections were detected in 67% (29/43) and 33% (14/43) of cases respectively.

#### Comparison between NGS and RLH

Using Cohen’s Kappa test, a perfect agreement between NGS and RLH was observed (κ = 0.83, 95% C.I. 0.60–1). Among the 29 cases of single infections, NGS genotyping results were in perfect agreement with the 24 samples genotyped by RLH. Data are summarized in S2 Table.

RLH and NGS methodology have both detected HPV16, HPV52, HPV6, HPV40, HPV70, HPV59, HPV55, HPV39, HPV31, HPV53, HPV68 types (Fig 4, S2 Table). NGS allowed the detection of types such as HPV6, 31, 70, 39, 42, 11, and 52, that were not detected by RLH despite the presence of their probes on the membrane. Five types were not among the probes used and were detected for the first time in a Tunisian study: HPV81, HPV74, HPV68, and HPV62 [13] (Fig 4, S1 Table).

The table below summarizes the differences between the 2 genotyping assays (Table 2).

**Table 2:** Summary of the comparison between NGS and RLH genotyping assays.

	Clinical samples	Unknown HPV	Detected types	Multiple infections	Single infection	Number of co-infection	HR-HPV	LR-HPV
RLH	43	12	14	7	24	4	11	3
NGS	43	0	25	14	29	6	12	13

HR-HPV, high risk HPV; LR-HPV, low risk HPV.

## Discussion

In this study, HPV genotyping was designed using a Nested PCR with PGMY and GP5 +/ 6+ followed by Miseq sequencing (Illumina platform). PGMY/GP+ had greater sensitivity and broader coverage of HPV genotypes compared to PYGMY alone, GP+ alone, MY11/09 (MY) alone or MY/GP [19–22].

The choice of the Illumina platform was based on the low number of read errors that can be generated (<0.4%), as well as its ability to generate short-reads that are more appropriate for HPV analysis. In addition, the preparation of the libraries for the Miseq platform did not require a large amount of DNA (4nM) [8].

Setting the NGS cut-off to 30 reads depth was based on recommendations from others studies [13]. For data analysis, the pipeline contained a step of assembly by Trinity software that was used to avoid the problem of samples containing mixed infections. In fact, small-sized reads can align with one region in the genome of another type, which causes false positives or over-estimation of one type over others [23].

In order to increase the throughput and reduce the cost of the NGS, a double indexing method was developed; it consisted in using primers with Miseq adaptor and indexes for libraries construction. This allowed genotyping of 86 samples (43 clinical samples + 43 proficiency test panel plasmids) in a single sequencing reaction. There are infinite possibilities of multiplexing since several index kits are available to combine indexes during libraries constructions. The study carried out by Xin Yi et al. (2014) [8] described a multiplexing protocol targeting 1170 samples in a single sequencing reaction. Multiplexing is an advantageous alternative in terms of cost effectiveness since the cost of the reagents required for a sequencing reaction is the most expensive component.

In this study, the results showed that NGS identified higher HPV numbers in the co-infections than RLH. Thus, the detection of multiple HR-HPV infection has become a key issue in the development of cervical lesions and the epidemiological status of the population by efficient genotyping method. In fact, the underestimation of the number of HPV types in patients who developed cervical lesions could underestimate the effect of multiple infections as a risk factor for persistent infections in healthy young women. Different studies have reported that multiple HR-HPV acted synergistically in cervical carcinogenesis [24, 25], and cancers with multiple HPV infections could be more resistant to therapy than those due to single infection [26]. Comparing the two genotyping approaches of clinical samples, we noticed the limit of RLH in comparison to NGS; RLH was less sensitive than NGS in detecting HPV31, HPV39, HPV84, HPV42, HPV68, HPV11 and HPV52. Some of these types are known to have an epidemiological relevance. In fact, HPV31 is one of the five most prevalent HPV types in high-grade cervical intraepithelial neoplasia and cervical cancer. It was recently added to a new nano-valent HPV vaccine [27, 28]. The lack of detection of these types by RLH may be due to a variant which was not covered by the probe or to a cross reactivity with another type present in the same sample. Meisal R et al., in a study of NGS based HPV genotyping reported a considerable variation in HPV type specificity between NGS and a DNA hybridization method [12].

Using NGS we identified new LR-HPV types that were not identified by RLH in the previous Tunisian study [13]: HPV81, HPV74, HPV43, and HPV62. Confirmation of these observations may be needed in cases where the types had low read numbers [12, 29]. This is not the case for the detected types in this study; the average numbers of reads of the new HPV types detected (HPV81, HPV43, HPV74, and HPV62) was considerably above the 30 reads depth cut-off. Cross-contamination between wells or tubes during extraction can also be a reason for observation of false positives especially in PCR based assays [12]. However, a low number of reads would confirm this hypothesis; it was not the case for the herein reported results.

The advantage of the detection of new types using NGS has been reported in other studies; Barzon L et al. [9] and Arroyo et al. [10] compared commercialized HPV genotyping kit to an NGS approach and identified by NGS new HPV types such as HPV74, HPV81, HPV53 and HPV62. NGS also detected a wider range of types in multiple infections and new variants [8–12, 30, 31]. In this context Conway et al. [32], genotyped HPV from oral samples demonstrated the ability of NGS technology to detect other HPV types that would not have been detected by traditional methods.

NGS identified novel LR-HPV (HPV81, HPV43, HPV62 and HPV74) that are considered as rare HPV types [33] and limited studies reported their prevalence. These types were not described so far in Tunisian studies neither in healthy women nor in women with precancerous and cancerous lesions [13, 34–36], which further indicates its relevance for epidemiology. Thus it will allow a more precise description of the HPV types distribution in a country. A more precise HPV genotyping will provide better correlation to the cytology and insights as regards their role in intra-epithelial cervical lesions.

## Conclusion

This is the first study of HPV genotyping using NGS in Tunisia to our knowledge. This approach was considerably more sensitive than the classical reverse line hybridization method. It was also able to identify novel HPV types that were not detected by RLH or other related methods in Tunisia. This can justify the use of such technique for an accurate assessment of the circulating genotypes for a better epidemiological apprehension. Following on this study, the developed NGS protocol will be used for HPV genotyping of all the clinical samples collected in the frame of an epidemiological study in Tunisia, variants evaluation, and phylogenetic analysis.

## Supporting information

S1 Table GP5+/6+ primers sequence coupled with Miseq adaptor (P5/P7). (DOCX)

S2 Table Genotyping results of the clinical samples using NGS and RLH. (XLSX)

## References

[1] Human papillomavirus vaccines: WHO position paper, May 2017. Releve epidemiologique hebdomadaire. 2017;92(19):241–68. 28530369

[2] Maglennon GA, Doorbar J. The biology of papillomavirus latency. The open virology journal. 2012;6:190–7 doi: 10.2174/1874357901206010190 23341854 PMC3547330

[3] de Villiers EM, Fauquet C, Broker TR, Bernard HU, zur Hausen H. Classification of papillomaviruses. Virology. 2004;324(1):17–27 doi: 10.1016/j.virol.2004.03.033 15183049

[4] Galan-Sanchez F, Rodriguez-Iglesias MA. Comparison of human papillomavirus genotyping using commercial assays based on PCR and reverse hybridization methods. APMIS: acta pathologica, microbiologica, et immunologica Scandinavica. 2009;117(10):708–15 doi: 10.1111/j.1600-0463.2009.02522.x 19775338

[5] Poljak M, Kocjan BJ. Commercially available assays for multiplex detection of alpha human papillomaviruses. Expert review of anti-infective therapy. 2010;8(10):1139–62 doi: 10.1586/eri.10.104 20954880

[6] Mori S, Nakao S, Kukimoto I, Kusumoto-Matsuo R, Kondo K, Kanda T. Biased amplification of human papillomavirus DNA in specimens containing multiple human papillomavirus types by PCR with consensus primers. Cancer science. 2011;102(6):1223–7 doi: 10.1111/j.1349-7006.2011.01922.x 21388488 PMC11159067

[7] Huang SL, Chao A, Hsueh S, Chao FY, Huang CC, Yang JE, et al. Comparison between the Hybrid Capture II Test and an SPF1/GP6+ PCR-based assay for detection of human papillomavirus DNA in cervical swab samples. Journal of clinical microbiology. 2006;44(5):1733–9 doi: 10.1128/JCM.44.5.1733-1739.2006 16672400 PMC1479179

[8] Yi X, Zou J, Xu J, Liu T, Liu T, Hua S, et al. Development and validation of a new HPV genotyping assay based on next-generation sequencing. Am J Clin Pathol. 2014;141(6):796–804 doi: 10.1309/AJCP9P2KJSXEKCJB 24838323

[9] Barzon L, Militello V, Lavezzo E, Franchin E, Peta E, Squarzon L, et al. Human papillomavirus genotyping by 454 next generation sequencing technology. Journal of clinical virology: the official publication of the Pan American Society for Clinical Virology. 2011;52(2):93–7 doi: 10.1016/j.jcv.2011.07.006 21802982

[10] Arroyo LS, Smelov V, Bzhalava D, Eklund C, Hultin E, Dillner J. Next generation sequencing for human papillomavirus genotyping. Journal of clinical virology: the official publication of the Pan American Society for Clinical Virology. 2013;58(2):437–42 doi: 10.1016/j.jcv.2013.07.013 23932809

[11] Meiring TL, Salimo AT, Coetzee B, Maree HJ, Moodley J, Hitzeroth, II, et al. Next-generation sequencing of cervical DNA detects human papillomavirus types not detected by commercial kits. Virology journal. 2012;9:164 doi: 10.1186/1743-422X-9-164 22897914 PMC3493284

[12] Meisal R, Rounge TB, Christiansen IK, Eieland AK, Worren MM, Molden TF, et al. HPV Genotyping of Modified General Primer-Amplicons Is More Analytically Sensitive and Specific by Sequencing than by Hybridization. PloS one. 2017;12(1):e0169074 doi: 10.1371/journal.pone.0169074 28045981 PMC5207713

[13] Ardhaoui M, Ennaifer E, Letaief H, Salsabil R, Lassili T, Chahed K, et al. Prevalence, Genotype Distribution and Risk Factors for Cervical Human Papillomavirus Infection in the Grand Tunis Region, Tunisia. PloS one. 2016;11(6):e0157432 doi: 10.1371/journal.pone.0157432 27299955 PMC4907453

[14] Eklund C, Forslund O, Wallin KL, Dillner J. Global improvement in genotyping of human papillomavirus DNA: the 2011 HPV LabNet International Proficiency Study. Journal of clinical microbiology. 2014;52(2):449–59 doi: 10.1128/JCM.02453-13 24478473 PMC3911320

[15] Gravitt PE, Peyton CL, Alessi TQ, Wheeler CM, Coutlee F, Hildesheim A, et al. Improved amplification of genital human papillomaviruses. Journal of clinical microbiology. 2000;38(1):357–61. doi: 10.1128/JCM.38.1.357-361.2000 10618116 PMC88724

[16] Bolger AM, Lohse M, Usadel B. Trimmomatic: a flexible trimmer for Illumina sequence data. Bioinformatics. 2014;30(15):2114–20 doi: 10.1093/bioinformatics/btu170 24695404 PMC4103590

[17] Clifford GM, Smith JS, Plummer M, Munoz N, Franceschi S. Human papillomavirus types in invasive cervical cancer worldwide: a meta-analysis. Br J Cancer. 2003;88(1):63–73 doi: 10.1038/sj.bjc.6600688 12556961 PMC2376782

[18] Hung JH, Weng Z. Mapping Short Sequence Reads to a Reference Genome. Cold Spring Harbor protocols. 2017;2017(2) doi: 10.1101/pdb.prot093161 27574204

[19] Cai YP, Yang Y, Zhu BL, Li Y, Xia XY, Zhang RF, et al. Comparison of human papillomavirus detection and genotyping with four different prime sets by PCR-sequencing. Biomedical and environmental sciences: BES. 2013;26(1):40–7 doi: 10.3967/0895-3988.2013.01.005 23294614

[20] Camargo M, Soto-De Leon S, Sanchez R, Munoz M, Vega E, Beltran M, et al. Detection by PCR of human papillomavirus in Colombia: Comparison of GP5+/6+ and MY09/11 primer sets. Journal of virological methods. 2011;178(1–2):68–74 doi: 10.1016/j.jviromet.2011.08.014 21884728

[21] Clifford GM, Vaccarella S, Franceschi S, Tenet V, Umulisa MC, Tshomo U, et al. Comparison of Two Widely Used Human Papillomavirus Detection and Genotyping Methods, GP5+/6+-Based PCR Followed by Reverse Line Blot Hybridization and Multiplex Type-Specific E7-Based PCR. Journal of clinical microbiology. 2016;54(8):2031–8 doi: 10.1128/JCM.00618-16 27225411 PMC4963525

[22] Karlsen F, Kalantari M, Jenkins A, Pettersen E, Kristensen G, Holm R, et al. Use of multiple PCR primer sets for optimal detection of human papillomavirus. Journal of clinical microbiology. 1996;34(9):2095–100 doi: 10.1128/jcm.34.9.2095-2100.1996 8862564 PMC229196

[23] Beerenwinkel N, Günthard HF, Roth V, Metzner KJ. Challenges and opportunities in estimating viral genetic diversity from next-generation sequencing data. Frontiers in microbiology. 2012;3:329 doi: 10.3389/fmicb.2012.00329 22973268 PMC3438994

[24] Wang L, Wang P, Ren Y, Du J, Jiang J, Jia X, et al. Prevalence of High-Risk Human Papillomavirus (HR-HPV) Genotypes and Multiple Infections in Cervical Abnormalities from Northern Xinjiang, China. PloS one. 2016;11(8):e0160698 doi: 10.1371/journal.pone.0160698 27494179 PMC4975475

[25] Trottier H, Mahmud S, Costa MC, Sobrinho JP, Duarte-Franco E, Rohan TE, et al. Human papillomavirus infections with multiple types and risk of cervical neoplasia. Cancer epidemiology, biomarkers & prevention: a publication of the American Association for Cancer Research, cosponsored by the American Society of Preventive Oncology. 2006;15(7):1274–80 doi: 10.1158/1055-9965.EPI-06-0129 16835323

[26] Munagala R, Donà MG, Rai SN, Jenson AB, Bala N, Ghim SJ, et al. Significance of multiple HPV infection in cervical cancer patients and its impact on treatment response. International journal of oncology. 2009;34(1):263–71. 19082497

[27] Beca F, Pinheiro J, Rios E, Pontes P, Amendoeira I. Genotypes and prevalence of HPV single and multiple concurrent infections in women with HSIL. Diagnostic cytopathology. 2014;42(11):919–23 doi: 10.1002/dc.23143 24623593

[28] Signorelli C, Odone A, Ciorba V, Cella P, Audisio RA, Lombardi A, et al. Human papillomavirus 9-valent vaccine for cancer prevention: a systematic review of the available evidence. Epidemiology and infection. 2017;145(10):1962–82 doi: 10.1017/S0950268817000747 28446260 PMC5974698

[29] Li T, Unger ER, Rajeevan MS. Universal human papillomavirus typing by whole genome sequencing following target enrichment: evaluation of assay reproducibility and limit of detection. BMC Genomics. 2019;20(1):231 doi: 10.1186/s12864-019-5598-0 30894118 PMC6425667

[30] Militello V, Lavezzo E, Costanzi G, Franchin E, Di Camillo B, Toppo S, et al. Accurate human papillomavirus genotyping by 454 pyrosequencing. Clinical microbiology and infection: the official publication of the European Society of Clinical Microbiology and Infectious Diseases. 2013;19(10):E428–34 doi: 10.1111/1469-0691.12219 23573945

[31] Nilyanimit P, Chansaenroj J, Poomipak W, Praianantathavorn K, Payungporn S, Poovorawan Y. Comparison of Four Human Papillomavirus Genotyping Methods: Next-generation Sequencing, INNO-LiPA, Electrochemical DNA Chip, and Nested-PCR. Annals of laboratory medicine. 2018;38(2):139–46 doi: 10.3343/alm.2018.38.2.139 29214758 PMC5736673

[32] Conway C, Chalkley R, High A, Maclennan K, Berri S, Chengot P, et al. Next-generation sequencing for simultaneous determination of human papillomavirus load, subtype, and associated genomic copy number changes in tumors. J Mol Diagn. 2012;14(2):104–11 doi: 10.1016/j.jmoldx.2011.10.003 22240447

[33] Co NNC, Chu L-O, Chow JKF, Tam JWO, Ng EKO. HPV Prevalence and Detection of Rare HPV Genotypes in Hong Kong Women from Southern China with Cytological Abnormalities. ISRN Virology. 2013;2013:312706 doi: 10.5402/2013/312706

[34] Hassen E, Remadi S, Chouchane L. [Detection and molecular typing of human papillomaviruses: prevalence of cervical infection in the Tunisian central region]. Tunis Med. 1999;77(10):497–502. 10670281

[35] Missaoui N, Hmissa S, Trabelsi A, Tahar Yacoubi M, Nouira A, Frappart L, et al. [Prevalence of HPV infection in precancerous and cancerous lesions of the uterine cervix in Tunisia]. Annales de biologie clinique. 2010;68(3):297–303 doi: 10.1684/abc.2010.0431 20478773

[36] Ennaifer E, Salhi F, Laassili T, Fehri E, Ben Alaya N, Guizani I, et al. Type-Specific Human Papillomavirus Distribution in Invasive Squamous Cervical Carcinomas in Tunisia and Vaccine Impact. Asian Pac J Cancer Prev. 2015;16(15):6769–72 doi: 10.7314/apjcp.2015.16.15.6769 26434909

