# Supplementary material for: Nested PCR followed by NGS: Validation and application for HPV genotyping of Tunisian cervical samples
Source: PLoS One. 2021 Aug 11;16(8):e0255914. doi: 10.1371/journal.pone.0255914 (PMC8357094; doi:10.1371/journal.pone.0255914)
Supplement: S1 Table — (DOCX) [file pone.0255914.s001.docx]

**S1 Table.** GP5+/6+ Primers sequence coupled with Miseq adaptor (P5/P7).

| **Primers** | **Sequence (5’-3’)** |
| --- | --- |
| **P5-GP5+** | **TCGTCGGCAG**CGTCAGATGTGTATAAGAGACAG TTT GTT ACT GTG GTA GAT ACCAC |
| **P7-GP6+** | **GTCTCGTGGG**CTCGGAGATGTGTATAAGAGACAG GAA AAA TAA ACT GTA AAT CAT ATT |
